# Effects of land use/cover on surface water pollution based on remote sensing and 3D-EEM fluorescence data in the Jinghe Oasis

**DOI:** 10.1038/s41598-018-31265-0

**Published:** 2018-08-30

**Authors:** Xiaoping Wang, Fei Zhang

**Affiliations:** 1 Key Laboratory of Smart City and Environmental Modeling of Higher Education Institute, College of Resources and Environment Sciences, Xinjiang University, Urumqi, 830046 People’s Republic of China; 2 Key Laboratory of Oasis Ecology, Xinjiang University, Urumqi, 830046 Xinjiang People’s Republic of China; 3 Engineering Research Center of Central Asia Geoinformation Development and Utilization, National Administration of Surveying, Mapping and Geoinformation, Urumqi, 830002 People’s Republic of China

## Abstract

The key problem in the reasonable management of water is identifying the effective radius of surface water pollution. Remote sensing and three-dimensional fluorescence technologies were used to evaluate the effects of land use/cover on surface water pollution. The PARAFAC model and self-organizing map (SOM) neural network model were selected for this study. The results showed that four fluorescence components, microbial humic-like (C1), terrestrial humic-like organic (C2, C4), and protein-like organic (C3) substances, were successfully extracted by the PARAFAC factor analysis. Thirty water sampling points were selected to build 5 buffer zones. We found that the most significant relationships between land use and fluorescence components were within a 200 m buffer, and the maximum contributions to pollution were mainly from urban and salinized land sources. The clustering of land-use types and three-dimensional fluorescence peaks by the SOM neural network method demonstrated that the three-dimensional fluorescence peaks and land-use types could be grouped into 4 clusters. Principal factor analysis was selected to extract the two main fluorescence peaks from the four clustered fluorescence peaks; this study found that the relationships between salinized land, cropland and the fluorescence peaks of C1, W2, and W7 were significant by the stepwise multiple regression method.

## Introduction

Water quality plays pivotal roles in habitat protection, agriculture, industry, and public health^1^. The sources of water quality pollution not only come from rivers and lakes of but also from the land use/cover and the production activities of humans^2,3^. Land use/cover is the carrier of human activity in watersheds. Landscape patterns control various biogeochemical and physical processes of watersheds. Therefore, one of the most significant consequences of landscape pattern change is the deterioration of river water quality^1^. Water pollution not only leads to the imbalance of river ecosystems but also threatens public health security and socio-economic sustainability^4^. To take effective measures for the protection of river water safety, a scientific interpretation of the relationship between land use/cover and river water quality is greatly needed.

Many previous studies have shown that land use/cover is a key factor affecting water quality pollution^5,6^. Some researchers reported relationships at catchment or watershed scales, while other researchers conducted studies at the riparian buffer scale^7,8^. Previous results have shown that land-use types are closely related to human activities, and there is a positive relationship between cropland and urban areas and water quality pollution indicators (e.g., nitrogen, phosphorus, ammonia) and a negative relationship between forests, sandy areas, and grasslands and water quality pollution indicators (e.g., nitrogen, phosphorus, ammonia)^9^. These previous analyses and approaches were all based on the assumption that relationships between water quality indicators and land-use patterns were constant over the entire study area. In recent years, optical tools such as fluorescence measurements have been developed as quick and general monitoring technologies for water quality^10–12^. In particular, excitation-emission matrix (EEM) spectroscopy can be used to extract the excitation and emission wavelengths of a wide range of water samples containing a variety of fluorescence peaks^13^. In fluorescence excitation-emission, there is a certain relationship between protein-like fluorescence peaks, including tryptophan and tyrosine, and the concentration of microbes, while humic-like fluorescence peaks can identify condensed humified organic substances^14,15^. The EEM technique with parallel factor analysis (PARAFAC), a three-way decomposition method, has been found to produce a useful diagnosis of fluorescence-independent spectral overlap components^16–18^. PARAFAC can identify different types of independent fluorescence spectra, and these can be separated by the high-resolution fluorescence imaging of the overlapping parts of several independent EEM components; thus, PARAFAC is capable of identifying even small changes.

The use of remote sensing images coupled with spatial analysis techniques to investigate the influence of land use/cover on surface water pollution has become a critical research topic. However, previous studies have focused on the correlation between water quality and land use/cover under fuzzy boundaries at catchment or watershed scales^19^. It is difficult to subdivide the watershed unit. Therefore, the studies on the correlation between water quality and land use/cover with quantitative boundaries are scare. In addition, studies on the response relationship between water quality and land use/cover at a small scale by radius methods are scare.

In this paper, we studied the arid area of the Jinghe Oasis, Central Asia, using three-dimensional fluorescence spectral and Gao Fen-1 (GF-1) satellite images, and then we combined the data with EEM-PARAFAC methods and self-organizing feature map neural network (SOM) methods to determine the connection between land use/cover and the fluorescence peaks. The objectives were: (1) identify the fluorescence peak of water bodies in the arid area; (2) analyze the relationship between land use/cover and the fluorescence peaks at multiple spatial scales; (3) examine the radius of action on the effect of land use/cover on surface water pollution; and (4) provide more information and references for the management and control of water pollution.

## Result and Analysis

### Land use/cover characteristics at various spatial scales

**Figure 1:**
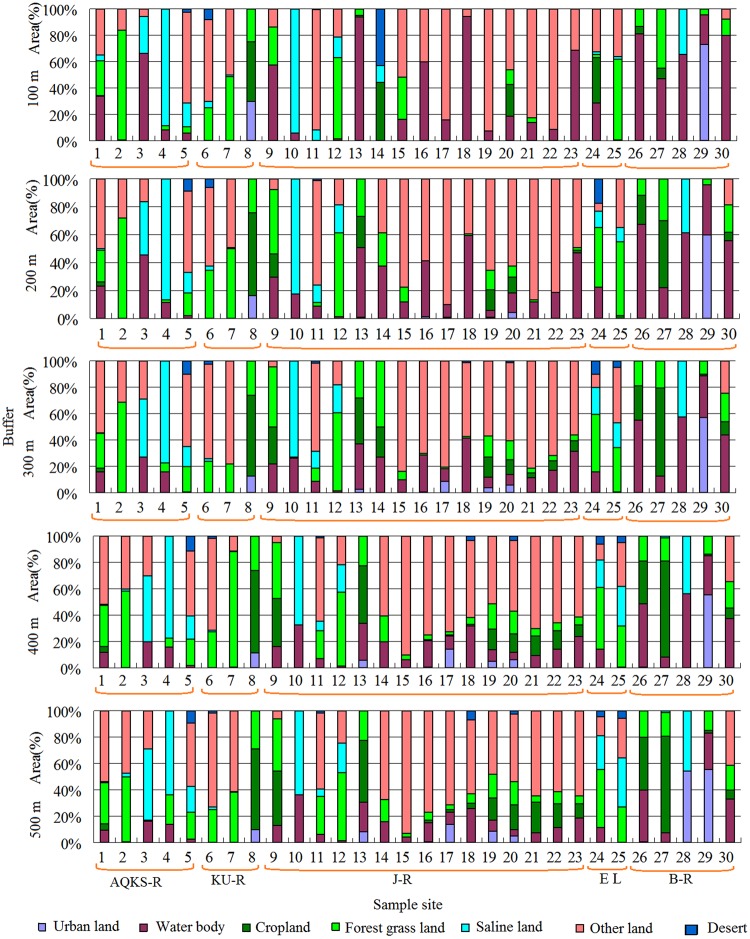
Statistical analysis of land use/covers area under different Radius (AQKS-R, represents Akeqisu River; KU-R represents Kuitun River; EL represents Ebinur Lake; B-R represents Bortala River) (Map by EXCEL (https://www.microsoft.com/software)).

To understand the differences in the land use/cover types between the river scale and the buffer zones, this study used the river scale and 100 m, 200 m, 300 m, 400 m and 500 m buffers (Figure 1) in the Jinghe Oasis, which includes the Bortala River (B-R), Jinghe River (J-R), Aqikesu River (AQKS-R) and Kuitun River (K–R). The percentage of urban land within the buffer zones declined slightly compared to the value at the river scale, with high values for sample 28 and sample 29, which were 56.2% and 48.2% under the 100 m and 200 m scale, respectively. The lowest values were for sample 25 and sample 34, which were 0% at the 100 m, 200 m, 300 m, 400 m and 500 m buffers. The proportion of other land in sample 16 and sample 17, however, reached up to 74.93% in the 100 m buffer scale, reflecting the existence of a large amount of undeveloped land within this area. This is the main feature of land use in arid areas. The proportion of salinized land in sample 3, sample 4 and sample 10 was as high as 60% at the 100 m buffer scale, and with an increase in the scale of the buffer, the value declined, reflecting the existence of a large amount of undeveloped land within this area.

### PARAFAC model components from EEM

**Figure 2:**
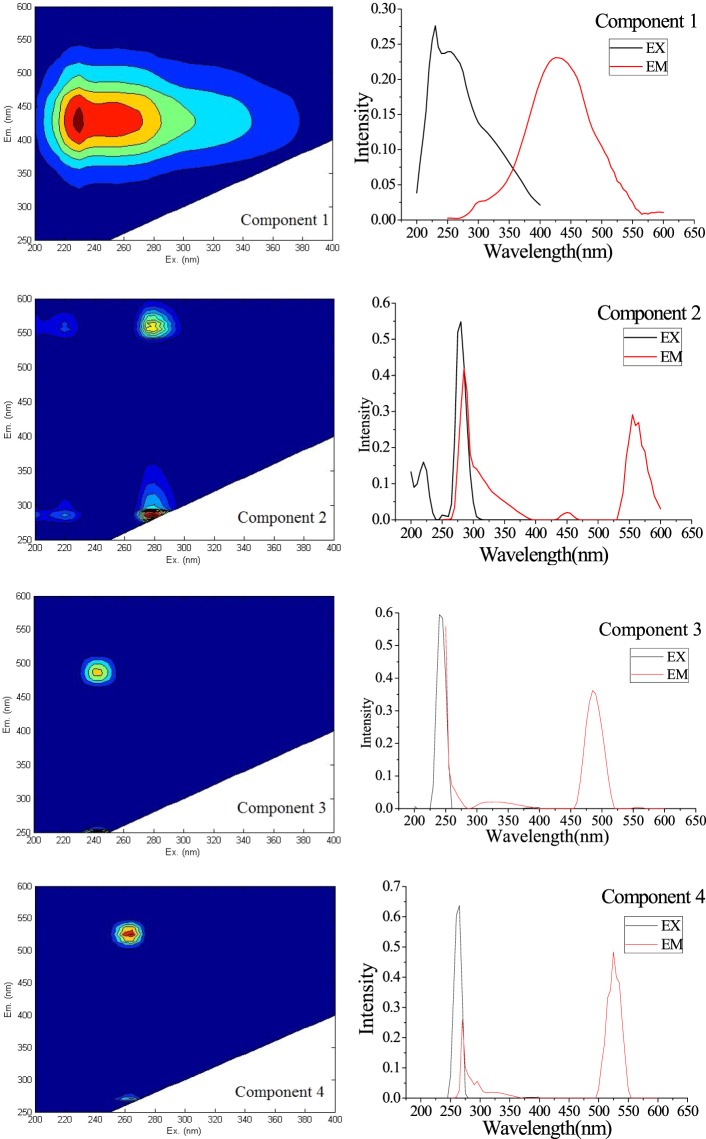
Contour plots of the four PARAFAC components decomposed from water samples of the Jinghe Oasis. (Map by Matlab (https://www.mathworks.com/software)).

Overall, all fluorescent EEM data were resolved into a successful PARAFAC model analysis. Figure 2 reveals each contour profile of the three PARAFAC components. Seven peaks were extracted, decomposed from water samples of the Jinghe Oasis, as shown in Table 1. However, W1 and W2 represent the peak of Raman scattering; therefore, they are not considered in further calculations.

**Table 1:** Peaks of the four PARAFAC components decomposed from water samples of the Jinghe Oasis.

Components	Peaks	EX(nm)	EM(nm)	Substance	Reference
C1	(C1)	255	425	Photodegradation product	^46^
C2	W1	215	280	—	—
	W2	275	550	humic-like	^47^
	W3	265	280	Photodegradation product	^48^
	W4	515	550	—	—
C3	W5	240	485	Humic substances + recent materials	^49^
C4	W6	260	265	—	—
	W7	260	485	tyrosine-like	^50^

**Figure 3:**
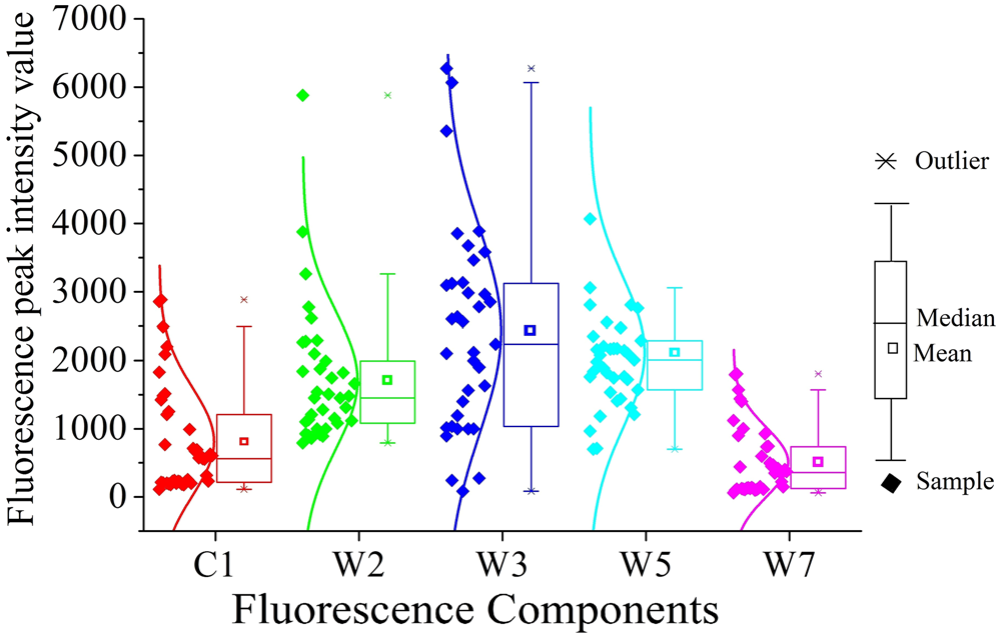
Fluorescence peak intensity value statistics (map by Origin 9.1 (http://www.originlab.com/software)).

Figure 3 shows the differences in C1, T2, T3, T5 and T7 at the Jinghe Oasis. C1, T2, T3, T5 and T7 represent photodegradation products, humic-like substances, photodegradation products, humic substances + recent materials and tyrosine-like substances, respectively. The value of C1 ranges between 179.2 and 2884, and the value of W1 ranges between 1013 and 9998. The high values of C1 and W1 suggest the photodegradation product content is different in the watershed. The values of W2 were the highest in sample 17, ranging from 791 to 9991. The values of W5 were the highest in sample 34, sample 35 and sample 36, ranging from 291 to 8991.

### Relationships between the fluorescence peak values and the land use and cover types

At small scales, a positive correlation indicates the influence of built-up land on the peak value of fluorescence, showing that, with the increase in the composition of urban land, the concentrations of these water quality parameters increase and make the contamination more serious (Table 2). It should be especially noted that C1 and W7 are influenced differently by urban land at 200 m scales, with R^2^ of 0.95. Petroleum is not affected by the composition of each land use type. C1 and W7 are influenced by salinized land at all scales, and the R^2^ increases as the scale increases. The R^2^ is 0.58 for the 200 m radius of operation. Therefore, relationships between the peak value of fluorescence and the land use/cover were explored within a 200 m radius of operation.

**Table 2:** Relationship between the peak values of fluorescence and the land use/cover types under different water radii of action.

Radius	components	Urban land	Water body	Cropland	Forest-grassland	Salinized land	Other land	Desert
100 m	C1	−0.088	−0.108	−0.204	−0.125	0.555^**^	−0.075	−0.062
	W2	0.019	0.177	0.03	−0.05	−0.413	0.008	−0.057
	W3	−0.06	0.262	−0.07	−0.003	−0.281	0.001	0.006
	W5	0.001	0.038	−0.063	−0.004	−0.187	0.147	−0.087
	W7	−0.087	−0.111	−0.211	−0.118	0.548^**^	−0.071	−0.053
200 m	C1	0.959^**^	−0.1	−0.246	−0.108	0.582^**^	−0.169	0.272
	W2	−0.345^*^	0.124	0.224	−0.068	−0.214	0.011	−0.044
	W3	−0.307^*^	0.194	0.15	−0.042	−0.3	0.068	−0.11
	W5	−0.194	−0.054	0.135	−0.039	−0.185	0.147	−0.073
	W7	0.932^**^	−0.104	−0.25	−0.099	0.571^**^	−0.164	0.275
300 m	C1	−0.085	−0.075	−0.287	−0.15	0.587^**^	−0.039	0.257
	W2	0.1	0.131	0.237	−0.098	−0.214	−0.049	−0.118
	W3	0.026	0.152	0.224	−0.064	−0.317	0.017	−0.159
	W5	0.111	−0.066	0.124	−0.065	−0.193	0.103	−0.116
	W7	−0.082	−0.079	−0.289	−0.146	0.575^**^	−0.032	0.255
400 m	C1	−0.108	−0.063	−0.265	0.127	0.535**	−0.146	0.238
	W2	0.182	0.145	0.201	−0.163	−0.209	−0.042	−0.17
	W3	0.084	0.11	0.191	−0.177	−0.339	0.109	−0.194
	W5	0.206	−0.061	0.107	−0.071	−0.209	0.081	−0.143
	W7	−0.103	−0.066	−0.267	0.129	0.524**	−0.138	0.237
500 m	C1	0.771**	−0.461**	−0.303	0.566**	0.510*	−0.549*	−0.109
	W2	0.482	0.336	0.666**	−0.125	−0.094	0.536*	0.208
	W3	−0.226	0.237	0.319	−0.133	−0.145	0.143	0.288
	W5	−0.023	0.012	0.338	−0.081	−0.198	0.041	−0.095
	W7	0.771**	−0.164	0.021	−0.017	−0.205	−0.242	−0.527**

**Correlation is significant at the 0.01 level. *Correlation is significant at the 0.05 level.

### The contribution of land use/cover to water quality pollution in a 200 m radius

#### Spatial framework of land use/cover in a 200 m radius

**Figure 4:**
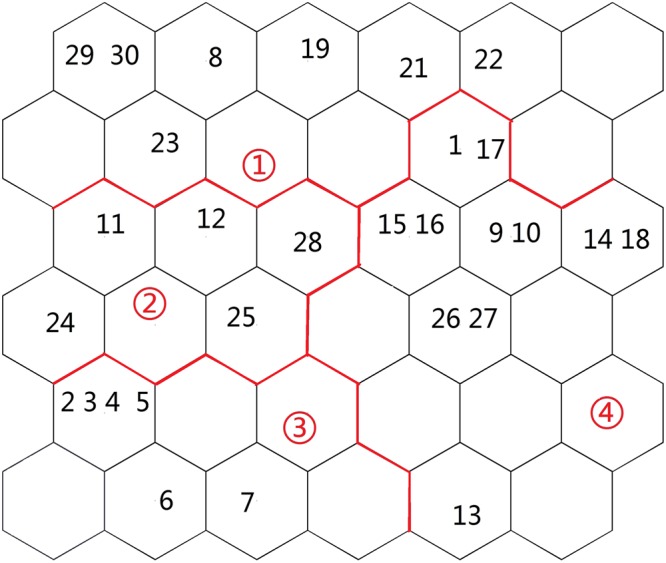
Sample distribution map of SOM analysis (Map by Matlab (https://www.mathworks.com/software)).

With regard to network structure selection, the neural network with a more complicated structure will generally have a better capability to address complicated non-linear problems. However, more complicated neural networks require a longer training time. Using a greater land use and cover type area can provide more abundant information; however, the correlation among indices will increase. The topological values were selected to determine grid size in this study, and the *k*-means clustering method was adopted to obtain the results. Overall, after the standard processing of the water quality data, the best network training effect was obtained from 36 (6 × 6) nerve cells (Figure 4), with QE and TE values of 1.033 and 0.001, respectively.

**Figure 5:**
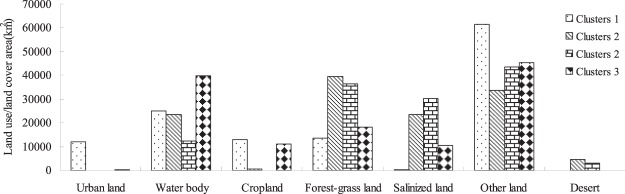
Average values for the land use/cover type area (map by Excel (https://www.microsoft.com/software)).

Figures 4–5 show that the distribution of the land use/cover type area varies in different clustering layers. Among the six clusters, the land use/cover type area is generally relatively better in Cluster 4. However, Cluster 2 and has 5 sampling points, 11, 12, 24, 25 and 28, which contain a water body. In addition, the water body, forest-grass land, cropland, salinized land, desert and other areas are low in Cluster 1, which indicates relatively concentrated human activity. Cluster 1 has 5 sampling points, 1, 17, 8, 19, 29, and 30. Cluster 3 has 6 sampling points, 2, 3, 4, 5, 6 and 7. Cluster 4 has 10 sampling points, 1, 17, 15, 16, 9, 10, 14, 18, 26 and 29.

**Figure 6:**
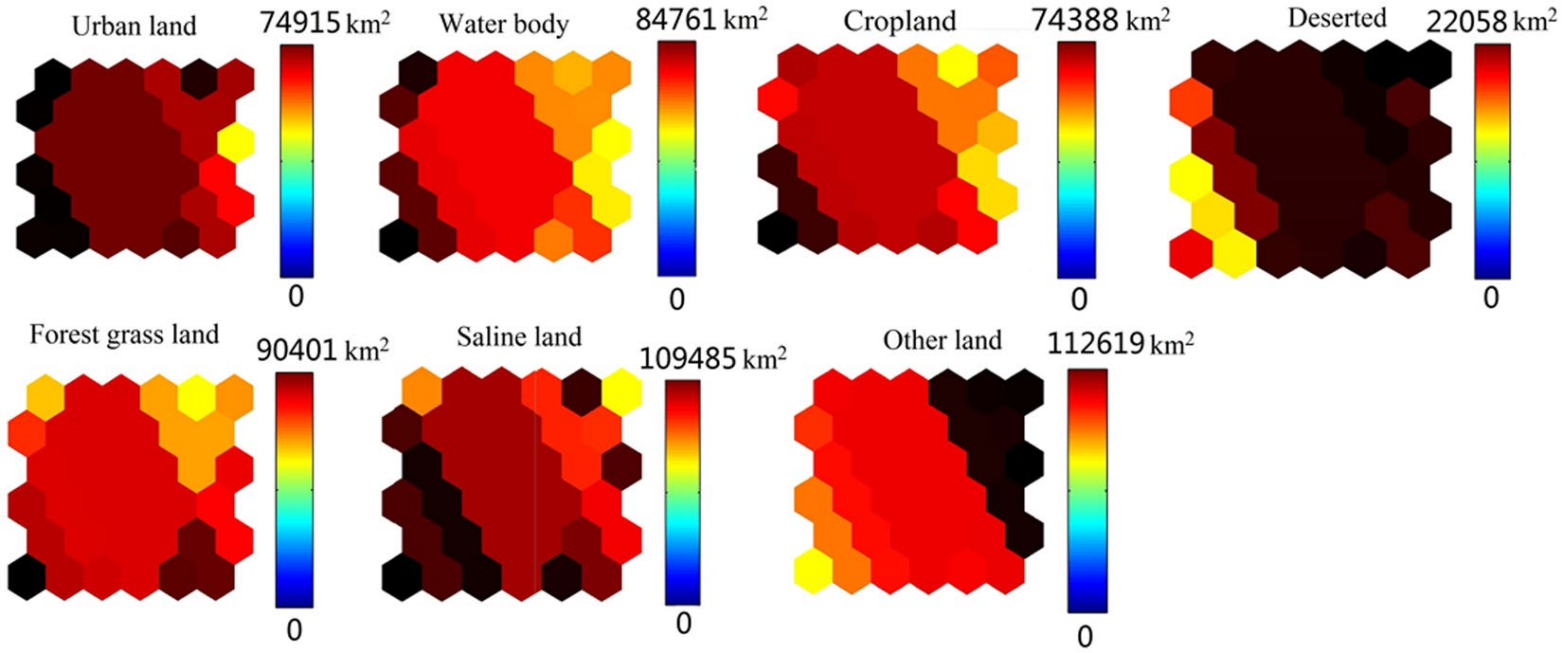
Visualization map of fluorescence values of components. (Map by Matlab (https://www.mathworks.com/software)).

Figure 5 shows the relations among the distribution of different variable classifications and water quality parameters. The land use and cover pattern and distribution of sampling points in Cluster1 are as follows: the town of TuoTuo is west of the sampling points, point 8 is in the middle of Jing River, points 19, 21, 22 and 23 are in the middle of Bortala River, and points 29 and 30 are in the eastern part of Bortala River. There is a large urban area in this region, so the cluster is therefore defined as urban land-oriented. The main samples of Cluster 2 were from the Bortala River and Jing River estuary, where forest-grassland and salinized land were the main land use types; thus, this cluster is defined as having grassland- and salinized land-oriented land use patterns. The rest of the samples were located near the farmland areas and were defined by a cropland-oriented utilization pattern. To further visualize the four components, the results are shown in Figure 6.

#### Spatial framework of the peak fluorescence value in the 200 m radius

**Figure 7:**
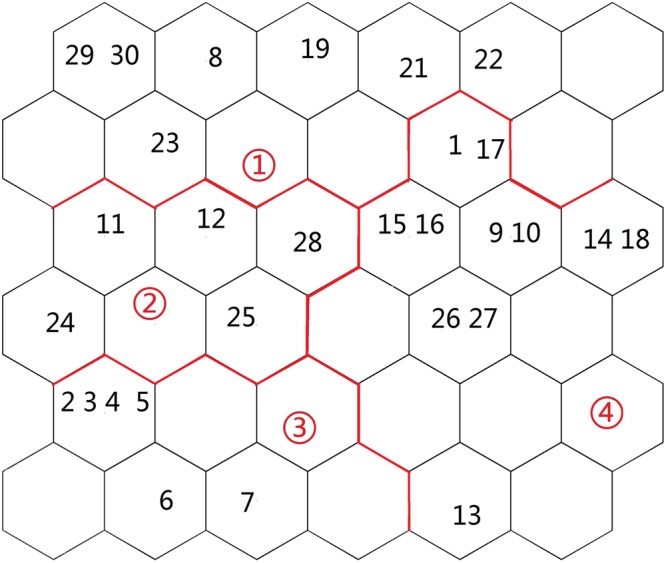
Sample distribution map of SOM analysis (map by Matlab (https://www.mathworks.com/software)).

Regarding network structure selection, the neural network with a more complicated structure will generally have better capability to address complicated non-linear problems. However, more complex neural networks require a longer training time. Using a greater peak value of fluorescence can provide more abundant information; however, the correlation among indices will increase. The topological values were selected to determine the grid size in this study, and the *k*-means clustering method was adopted to obtain results. Overall, after the standard processing of the water quality data, the best network training effect was obtained from 36 (6 × 6) nerve cells (Figure 7), and the QE and TE values were 1.043 and 0.001, respectively.

**Figure 8:**
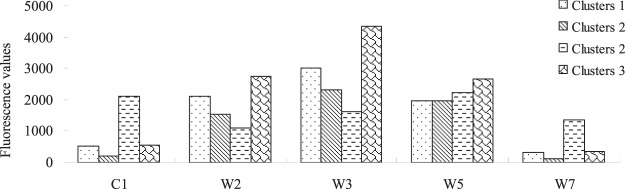
Average values for the peak value of fluorescence (map by EXCEL (https://www.microsoft.com/software)).

Figures 7–8 show that the distribution of the peak fluorescence value varies in different clustering layers. Among the four clusters, the peak fluorescence value distribution is generally relatively better in cluster 4. However, Cluster 2 only has 4 sampling points, 11, 12, 24, 25 and 28, which contain a water body. Cluster 1 has 5 sampling points, 1, 17, 8, 19, 29, and 30. Cluster 3 has 6 sampling points, 2, 3, 4, 5, 6 and 7. Cluster 4 has 10 sampling points, 1, 17, 15, 16, 9, 10, 14, 18, 26 and 29.

**Figure 9:**
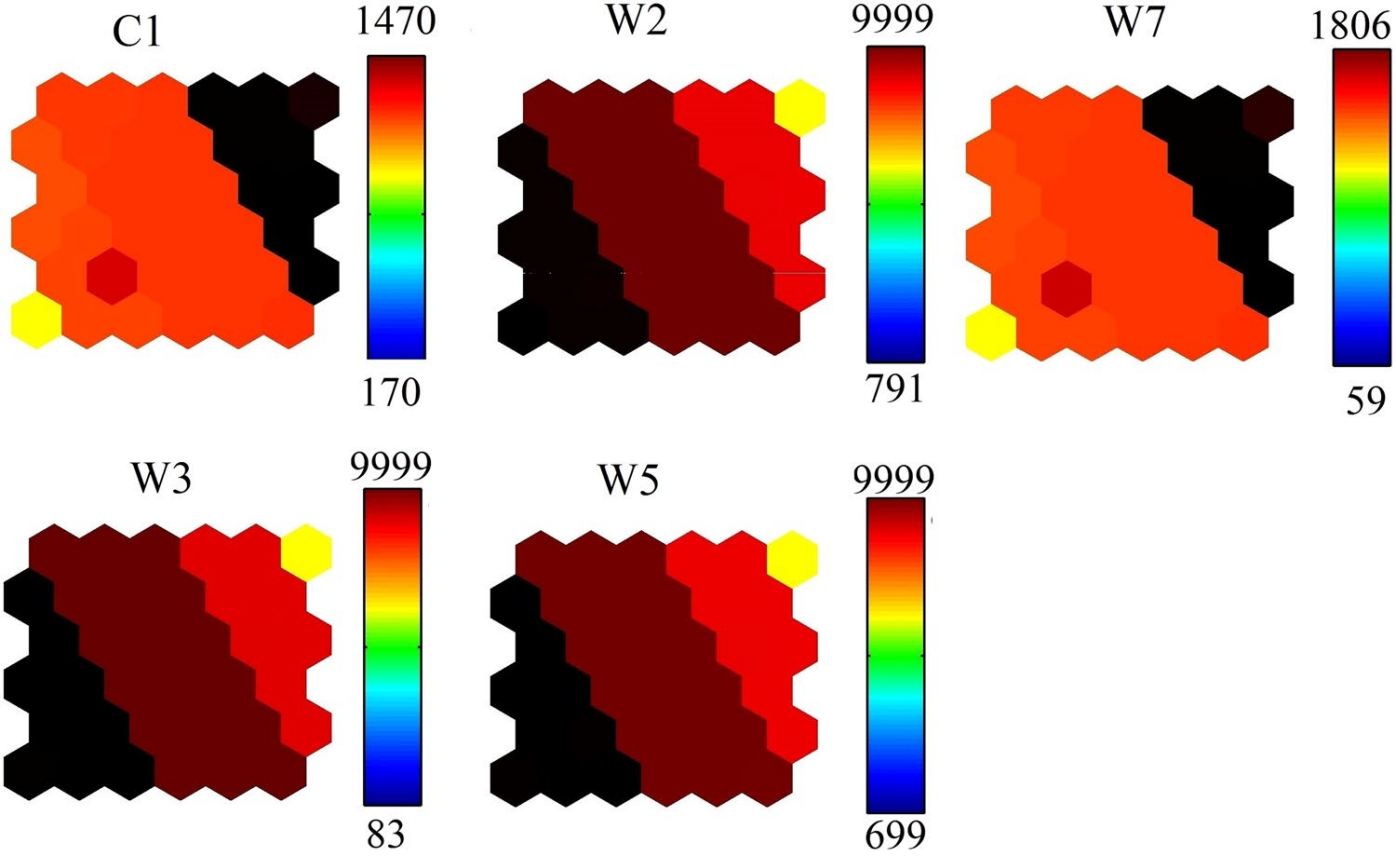
Visualization map of the peak values of fluorescence (map by Matlab (https://www.mathworks.com/software)).

Figure 8 shows the distribution relation among the various variables and water quality parameters of different clusters. For example, C1, W2 and W7 are recorded in the right corner of the SOM network, thereby indicating a declining trend in the southern part of Ebinur Lake and the surrounding Kuitun River. The main samples of cluster 2 were from the Bortala River and the Jing River, which flow into the lake. Cluster 3 is mainly a class of six samples from the Akeqisu River and the Kuitun River. The rest of cluster 4 is located near farmland. To further visualize the four components, the results are shown in Figure 9.

#### Principal components of fluorescence spectra under different clusters

Data redundancy in fluorescence data is often important for spectral analysis. The principal component analysis method was used to reduce the dimension of data in this study. Three principal components were selected that accounted for a cumulative variance greater than 85%. The main components of the band and the main score matrix are shown in Table 3.

**Table 3:** Surface water fluorescent components score matrix in the Jinghe Oasis.

Types	Cluster 1			Cluster 2			Cluster 3			Cluster 4		
	1	2	3	1	2	3	1	2	3	1	2	3
C1	−0.262	**0.960**	−0.096	0.693	0.517	0.458	−0.964	0.180	0.166	−0.701	0.703	−0.024
W2	0.879	0.467	−0.088	**0.952**	−0.161	0.129	**0.995**	0.073	−0.061	**0.861**	0.481	0.000
W3	**0.976**	0.008	0.186	−0.504	0.828	−0.212	0.883	0.099	0.454	**0.902**	0.311	−0.092
W5	0.903	0.148	0.203	0.908	0.11	−0.083	0.824	0.562	−0.052	0.816	0.508	0.084
W7	−0.232	0.961	−0.133	0.028	**0.993**	−0.052	−0.977	0.115	0.153	−0.695	0.706	−0.043

#### The contribution of land use/cover to water quality pollution in a 200 m radius

Using the multiple regression method, the contribution of land use/cover type to the fluorescence peak of the water body area was analyzed. R^2^ and F values were used to test their correlations, as shown in Table 4. At cluster 1, the regression analysis between the land use/cover area and the principal components of the fluorescence spectra shown that there is no significant relationship between W7, W2 and the land type; similarly, there is no significant relationship between C1 and the corresponding clustering component of the land use, the contribution of salinized land and forest-grassland types (R^2^ = 0.91). At cluster 2, the regression analysis between the land use/cover area and the principal components of the fluorescence spectra shows there is a significant relationship between W2 and the urban land and cropland, with R^2^ of 0.49 and 0.67, respectively. At cluster 3, the regression analysis between the land use/cover area and the principal components of the fluorescence spectra shows that there is no significant relationship between W2 and the desert or salinized land, with R^2^ of 0.69. At cluster 4, the regression analysis between the land use/cover area and the principal components of the fluorescence spectra shows there is no significant relationship between W2 and the salinized land and “other” area (R^2^ is 0.66). Salinized and construction land types are major contributors to the dissolved organic matter affecting the surface water quality in the Jinghe Oasis.

**Table 4:** Contribution of land use/cover to the fluorescence peaks of dissolved organic matter in surface water.

Cluster	y	X (Area)	Model	R^2^	F	SD
1	C1	Salinized land, Forest-grassland	y = 0.002x_Salinized land_ − 0.008x_Forest-grassland_ + 734.46	0.91	3.02	59.8
2	W2	Urban land, Cropland	y = 0.006x_Urban land_ − 0.008x_Cropland_ + 408.69	0.67	3.11	57.2
2	W7	Urban land, Cropland	y = −0.001x_Urban land_ − 0.005x_Cropland_ + 121.28	0.49	1.46	11.8
3	W2	Desert, Salinized land	y = 00.063x_Desert_ − 0.007x_Salinized land_ + 1591.4	0.69	3.39	269.1
4	W3	Salinized land, Other land	y = 0.006x_Other land_ − 0.03x_Salinized land_ + 4402.79	0.66	2.98	70.2

## Discussion

### The water environment is reflected by the fluorescence peak

Intensive land use in river watersheds and the rapid response of organic pollutants from different sources may cause the substantial deterioration of water quality, posing a direct or indirect threat to the quality of life of local people and the health of aquatic ecosystems^14,20^. Excitation-emission matrix (EEM) spectroscopy can be used to interpret a wide range of excitation and emission wavelengths contained within a variety of fluorescing water samples. In excitation-emission fluorescence, there is a relationship between fluorescence peaks and the number of water quality parameter^21^. The fluorescence peak of a water body reflects its environmental conditions. Research by Wang *et al*., indicates that 3D-fluorescence techniques are capable of estimating and monitoring surface water pollution in the Jinghe Oasis^14^. Fluorescence spectroscopy has received much attention in recent years due to its potential application in monitoring the water of rivers and lakes. This technique is attractive for monitoring the water quality in inland water bodies, as it is a rapid technique that requires no reagents and no sample preparation for analysis. The relationship between water quality and land use/cover is equivalent to the relationship between the peak of fluorescence and land use/cover. Kiedrzyńska *et al*. found that cropland areas were found to influence nitrogen, and forests areas were negatively related to loads of both nitrogen and phosphorus^19^, important organic matter factors in this study. Therefore, the results of this study are consistent with previous results.

### The influence of spatial and temporal scales

The influence of land use/cover patterns on water quality fluorescence is scale dependent^22^. The results showed that the 200 m action radius was better than the other action radii in explaining the overall fluorescence variations. The 200 m action radius acts as a filter by reducing surface runoff, processing nutrients to improve the water quality of rivers^23–26^. The radius of action was first introduced to explore the effect of land use on water quality in arid areas. Although it is essentially a case of buffer analysis, it also provides a new way of thinking about the effect of land use on surface water quality. The radius of action is based on the analysis of geospatial data, which lacks a strong theoretical basis, the primary problem to be addressed by future research.

### Management suggestions

The urban areas and salinized land in this watershed were mainly dispersed along the river, which had a negative effect on the river water quality. Consistent with a typical continental climate, this region is extremely dry and windy, and it has little rainfall and frequent dust storms in the Jinghe Oasis. Therefore, it is important to control urban runoff and ensure that the water quality meets national standards. Salinized land had a strong impact on water quality at a large scale. Reasonable irrigation and soil salt improvements are important measures. Forest-grassland areas have strong contributions to water quality variations at the river scale. Landscape pattern planning should be used to improve the water quality of watersheds in the arid region of central Asia.

## Conclusion

In the scope of applied conservation, understanding the impact of the surrounding land use/cover and human activities on the water quality at multiple scales is essential to adapt scale appropriate strategies to protect and rehabilitate in basin scale. The influence of the landscape on the water quality is scale dependent; this scale is beneficial to the management of water quality, convenience, economy and safety. Therefore, the key problem of the effective management of surface water is identifying the effective radius of the surface water pollution and blocking the pollution source. The Jinghe Oasis is located in the China-Kazakhstan border in the Xinjiang Uyghur Autonomous Region of China; we demonstrated the potential of integrated remote sensing and three-dimensional fluorescence technologies to investigate the effect of land use/cover types on surface water. The PARAFAC model and self-organizing map (SOM) neural network model were used to determine the effects of land use/cover types on water quality.

1. The four fluorescence components that were successfully extracted by the PARAFAC factor analysis modeling from the fluorescence EEM data are as follows: microbial humic-like (C1), terrestrial humic-like organic substances (C2, C4), and protein-like organic substances (C3).
2. Taking 30 water sampling points to build 5 buffer zones (100 m, 200 m, 300 m, 400 m, and 500 m), we found that “the most significant relationship between land use type and fluorescence components is found with 200 m radios, and the maximum contribution is from buildup land and salinized land.
3. Typical three-dimensional fluorescence peak and land use type were classified by the SOM neural network method, which demonstrated that four different types exist between three-dimensional fluorescence peak and land use types.
4. A principal factor analysis method applied to four fluorescence peaks and the stepwise multiple regression method showed that the clustering type contributes mostly to the surface water organic pollution in Ebinur lake were salinized land and cropland land, which was the contributed source of C1, W2, W7 fluorescence peak; salinized land was the most contributed source of W2, W3 fluorescence peak.

The relationship between the land use/cover and the water quality is complicated and can be influenced by numerous factors. From the perspective of the landscape, the design of effective radius of the surface water pollution is not simply related to the land use types, but it also depends on the spatial structure of the land use types. Therefore, the effective radius can better solve the water quality management in the watershed.

## Materials and Methods

### Study area

**Figure 10:**
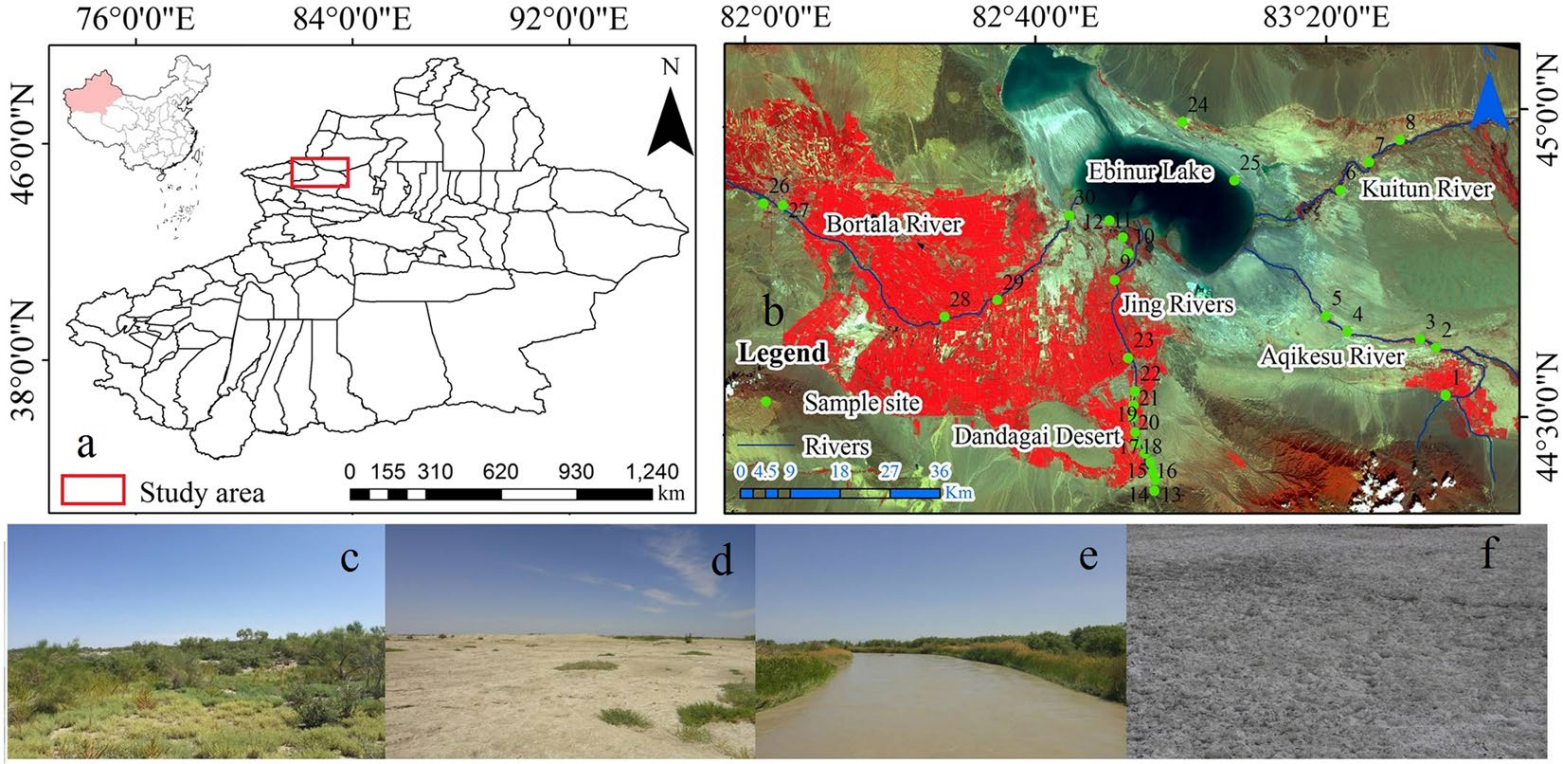
(**a**) Map of the study area with an inset map showing the location of the Xinjiang Autonomous Region in China; (**b**) satellite map of the study area; (**c**) forest-grassland, (**d**) other land, (**e**) water body, (**f**) salinized land (photographed (**c**–**f**) by Xiaoping Wang (map of (**a**,**b**) by ArcGIS10.2.2 (http://www.esri.com/software/arcgis)).

The Jinghe Oasis is located in the center of Eurasia in the northwest Xinjiang Uygur Autonomous Region at 44°02′∼45°10′N and 81°46′∼83°51′E. The Jinghe Oasis is composed of wetland and desert oasis vegetation and wildlife and is a national desert ecological reserve. The study area has a unique wetland ecological environment, and it has been listed as the Xinjiang Uygur Autonomous Region “Wetland Nature Reserve” (Figure 10). The Jinghe Oasis was once fed by 12 branch rivers belonging to three major river systems, and the major rivers were the Bortala River (B-R), Jinghe River (J-R), Aqikesu River (AQKS-R) and Kuitun River (K-R). Owing to natural environmental changes and human activities (i.e., modern oasis agricultural development), many rivers gradually lost their hydraulic connections with Ebinur Lake, and only the Bortala River and Jinghe River now supply water to Ebinur Lake. The western region of the Bortala River (B-R) valley is south of the Jing River (J-R) oases and the Dandagai Desert and east of the Mutetaer desert zone of the lower reaches of the Akeqisu–Kuitun River (AKQS-R). Ebinur Lake is located in the center of a watershed at the lowest elevation and represents a typical lake of the arid areas of Central Asia. The total watershed area is 50,621 km^2^. It is surrounded by a mountainous region (24,317 km^2^; Alatau Mountains) and plain areas (26,304 km^2^) to the north, west and south^27,28^. The climate is a typical temperate arid continental climate, and the mountain–oasis–desert system has typical temperate arid ecological characteristics. The study region is located inland (2000 km away from the Pacific and Indian Ocean and 3000 km away from the Arctic Ocean); the moisture sources in the study area are derived from the Atlantic Ocean (7000 km), but overall, there is limited water vapor transport from maritime areas^29^.

### Data sources and Data processing

#### Water sample acquisition and processing

Water samples were collected on 5 July 2016 from 29 locations within the Jinghe Oasis, a typical arid oasis. The collected samples were kept in low-temperature cold storage (under 2 °C) during transport before the water quality measurements were carried out in the laboratory. Samples were transported in polyethylene plastic bottles, previously washed in 10% HCI and cleaned with deionized water, to minimize changes in the water chemical characteristics.

The collected samples were filtered using a pre-washed GF/F filter. All fluorescence intensities were determined using a Cary Eclipse Fluorescence Spectrophotometer (F-7000, Hitachi High-Technology Corp, Tokyo, Japan). The spectrophotometer that measured the fluorescence spectra was equipped with a 150 W xenon arc lamp as the light source and two grating monochromators coupled with a slit as the EEM wavelength selectors. The scanning speed was set to 60000 nm/min. Therefore, each measurement period was 2 minutes in duration. The EEMs were measured every 10 nm over an excitation range of 200–450 nm, with an emission range of 200–550 nm by 10 nm. MilliQ water EEMs were used as blanks and subtracted from each sample EEM. The emission and excitation correction files generated by the FluoroMax manufacturer were applied to each MilliQ-subtracted sample EEM. Fluorescence intensities were standardized to a Raman peak at 395 nm emission, as suggested by Lawaetz^30^.

#### Remote sensing data

Medium spatial resolution cloud-free GF-1 images were used in this study (Table 5) and were acquired near the actual water sampling date on April 12th, 2016. These data were obtained from CRESDA (China Resources Satellite Application Center, http://www.cresda.com/CN/). The images were acquired in clear and dry weather conditions during the dry season. These GF-1 satellite images have a ground sampling distance (GSD) of 16 m and a pan band GSD of 8 m. The GF-1 images contain 5 bands that record the reflected or emitted radiation from the Earth’s surface in the B, G, R NIR and pan bands of the electromagnetic spectrum.

**Table 5:** Image information for GF-1.

Band	Reflectance	Resolution
1	Blue (0.45–0.52) um	16
2	Green (0.52–0.59) um	16
3	Red (0.63–0.69) um	16
4	NIR (0.77–0.89) um	16
5	PAN (0.52–0.89) um	8

The sensor and atmosphere can cause changes to the spectral characteristics of a target in a multitemporal remote sensing image, which affects the extraction of image data^31^. Therefore, the remote sensing images must undergo an atmospheric correction. To accomplish this correction, the pre-processing of GF-1 images was first conducted using ENVI 5.1 (Environment for Visualizing Images 5.1) (ExelisVisual Information Solution Corporation, America) software. Universal Transverse Mercator (UTM) projection was selected to rectify the satellite images. The GF-1 images were also geometrically corrected with a previously corrected GF-1 image that had a geometric accuracy of <0.5 pixels^32^. Then, the ENVI 5.1 radiometric calibration tool and the gain and deviation ratio in the GF-1 image data head document were used for the radiometric calibration of GF-1 data. Finally, the ENVI5.1 FLAASH atmospheric correction model was used for the atmospheric correction of the remote sensing images.

### Methods

The methodology is explained in the following section, with a conceptual flow chart describing the methodology. Figure S1 shows the workflow of the study detailed in the following sections.

#### Data Fusion

Data fusion is useful because it takes advantage of different spectral and/or resolution information for effective image interpretation. Pan bands and four multispectral band images were used for the image fusion^33^. We implemented selective principal component analysis (S-PCA) transformation, rather than the conventional standard PCA method.

#### Land use land cover clustering based on decision tree classification

The decision tree (DT) classifier is a simple and widely used classification technique. The DT classifier is an effective method to incorporate a variety of data types from multiple sources to find pixels that fulfill the criteria^34–36^. We conducted radiation and orthographic corrections for the remote sensing image data combined with 1:50,000 digital elevation model (DEM) data. We established five land use/cover types by using the Environment for Visualizing Images software (ENVI Version 5.0), urban land, cropland, forest-grassland, water body, salinized land, desert and others based on the actual conditions of the research zone. The final results showed that the producer’s accuracy of the classified LCLUC maps was 85.29%. The user’s accuracy of the classified LCLUC maps was 84.47%. The overall accuracy was 89%, and the kappa coefficient was 0.88.

#### PARAFAC Modeling

PARAFAC applications allow full use of the fluorescence EEM data samples. Fluorescence spectrum data multiplexer (three-way) as a sample of the fluorescent changes depends on the wavelength of light absorption (excitation) and fluorescence wavelength was observed (emission). PARAFAC decomposes the EEM dataset into a set of trilinear terms and a residual array^37^ and fits an equation by minimizing the residual sum of squares of three linear models as follows:

1 $${x}_{ijk}=\sum _{f=1}^{F}{a}_{if}{b}_{if}{c}_{kf}+\varepsilon {i}_{jk},\,\,i=1\ldots {\rm{I}};j=\ldots .J;\,{\rm{K}}=1\ldots {\rm{K}}$$

where x_ijk_ is the intensity of the three-way data array for the *i*th sample at the emission wavelength *j* and excitation wavelength *k*; *a*_*if*_ is directly proportional to the concentration of the *f*th three-way data array in the *i*th sample (defined as scores); *b*_jf_ and *C*_*kf*_ are the estimates of the three-way data; *F* represents the number of components in the model; and *e*_ijk_ is the residual element, representing the variability not accounted for by the model^38,39^.

MATLAB 2014a (MathWorks, Natick, MA, USA) and the DOM Fluor toolbox (http://www.models.life.ku.dk) were selected to perform the complete PARAFAC modeling. Residual analysis, spectral scores at the core of the consistency and visual inspection of each group were selected to diagnose the correct number of components^37^. The fluorescence intensity of each component (${I}_{i}$) was estimated using the following formula^40^:

2 $${I}_{i}=Scor{e}_{s}\times E{x}_{i}(\lambda \,\max )\times E{m}_{i}(\lambda \,\max )$$

where $Scor{e}_{s}$ represents the *i*th relative fluorescence intensity; $E{x}_{i}(\lambda \,\max )$ represents the first *N* components to stimulate the maximum load; and $E{m}_{i}(\lambda \,\max )$ represents the first n components of the emission load of the maximum number.

#### Analysis of water pollution radius

The “radius of action” was first introduced in the engineering field to discuss the quantity of blasting and the accurate blasting scope in the multiboundary blasting system. The term “radius of water quality action” was introduced to understand the effective range of land use in watersheds. To summarize the results of previous studies, we explored the effect of land use at a range of scales, 100 m, 200 m, 300 m, 400 m to 500 m, on the water quality in rivers. The framework is designed as shown in Figure S2.

#### Recognition of land use/cover and fluorescent component spatial characteristics based on the self-organizing map SOM method

A self-organizing map (SOM) is one of the branches of artificial neural network algorithms. It is a self-organizing and self-learning network visual method that can express multidimensional spatial data in low-dimensional points through non-linear mapping^41^. The SOM is an all-purpose classification tool that can connect samples with variables^42^. In recent years, the SOM has become increasingly popular in environmental research because of its capacity to address non-linear relations. The idea motivating the development of the SOM method was to represent a large amount of data typical of samples. The map usually has a 2D structure with a map unit associated with a weight vector.

3 $${N}_{ij}=\{{W}_{ij};1\le i\le L,1\le {J}\le M\}$$

where *N*_*ij*_ is a 2D map grid (also called a neuron); *W*_*ij*_ is the weight vector assigned to (i, j), the unit of SOM architecture; and *L* and *M* are number of rows and columns, respectively^43–45^. The steps of the SOM algorithm are displayed as follows:

- **Step 1:** Data normalization and SOM network initialize, the weight vector *w*_*ij*_ (*i* = *1*, *2*, *……*, *S; j* = *1, 2, 3, ……, R*) is randomly set in the interval [0, 1], *R* is the sample dimension, and *S* is the number of output neurons.
- **Step 2:** An input vector ${p}_{k}=({p}_{1}^{k},{p}_{2}^{k},\mathrm{......}{p}_{R}^{K})$ (*k* = *1, 2… M*, where *M* is the number of samples) is presented to the SOM network, and distance is calculated.
- **Step 3:** The smallest distance is chosen, and the best-matching unit (BMU) is identified.
- **Step 4:** The weight vector *w*_*ij*_ in the neighbor ration is updated.
- **Step 5:** The process proceeds in an iterative way until the optimal number of iteration steps is satisfied, and then it returns to step 2.

The SOM technique has a distinct capability to represent the complex relationships of the fluorescence intensity data and the land use area data using component planes and U-matrix. All simulations were implemented in MATLAB R2014a using an SOM toolbox.

### Statistical Analyses

A descriptive statistical analysis was applied to evaluate the water quality indices and EEM-PARAFAC and 3D Fluorescence spectral index. Linear regression and correlation analyses were constructed using Origin 8.0 (OriginLab Corporation, America). The significances of the correlations in the statistics were evaluated using *P* values and *t* values.

## Electronic supplementary material

Supporting information
