# Supplementary material for: Effects of land use/cover on surface water pollution based on remote sensing and 3D-EEM fluorescence data in the Jinghe Oasis
Source: Sci Rep. 2018 Aug 30;8:13099. doi: 10.1038/s41598-018-31265-0 (PMC6117340; doi:10.1038/s41598-018-31265-0)
Supplement: Supplementary file 1 — Supporting information [file 41598_2018_31265_MOESM1_ESM.doc]

**Supplementary material**

**Effects of land use/cover on surface water pollution based on remote sensing and 3D-EEM fluorescence data in the Jinghe Oasis**

**Xiaoping WANG ^a,b^ Fei ZHANG ^a,b,c^**

^a^. Key Laboratory of Smart City and Environmental Modeling of Higher Education Institute, College of Resources and Environment Sciences, Xinjiang University, Urumqi, 830046, People’s Republic of China

^b^. Key Laboratory of Oasis Ecology, Xinjiang University, Urumqi, 830046 Xinjiang, People’s Republic of China

^c^. Engineering Research Center of Central Asia Geoinformation Development and Utilization, National Administration of Surveying, Mapping and Geoinformation, Urumqi 830002, People’s Republic of China

Corresponding author: Fei ZHANG Tel:13579925126 E-mail:zhangfei3s@163.com

**Supporting information legends**

**Fig. S1|** Conceptual model for the methodology (Map by Visio (https://www.microsoft.com/software))

**Fig. S2|** Map of the buffer radius (Map by ArcGIS10.2.2 (http://www.esri.com/software/arcgis))

Fig.S1 Conceptual model for the methodology (Map by Visio (https://www.microsoft.com/software))


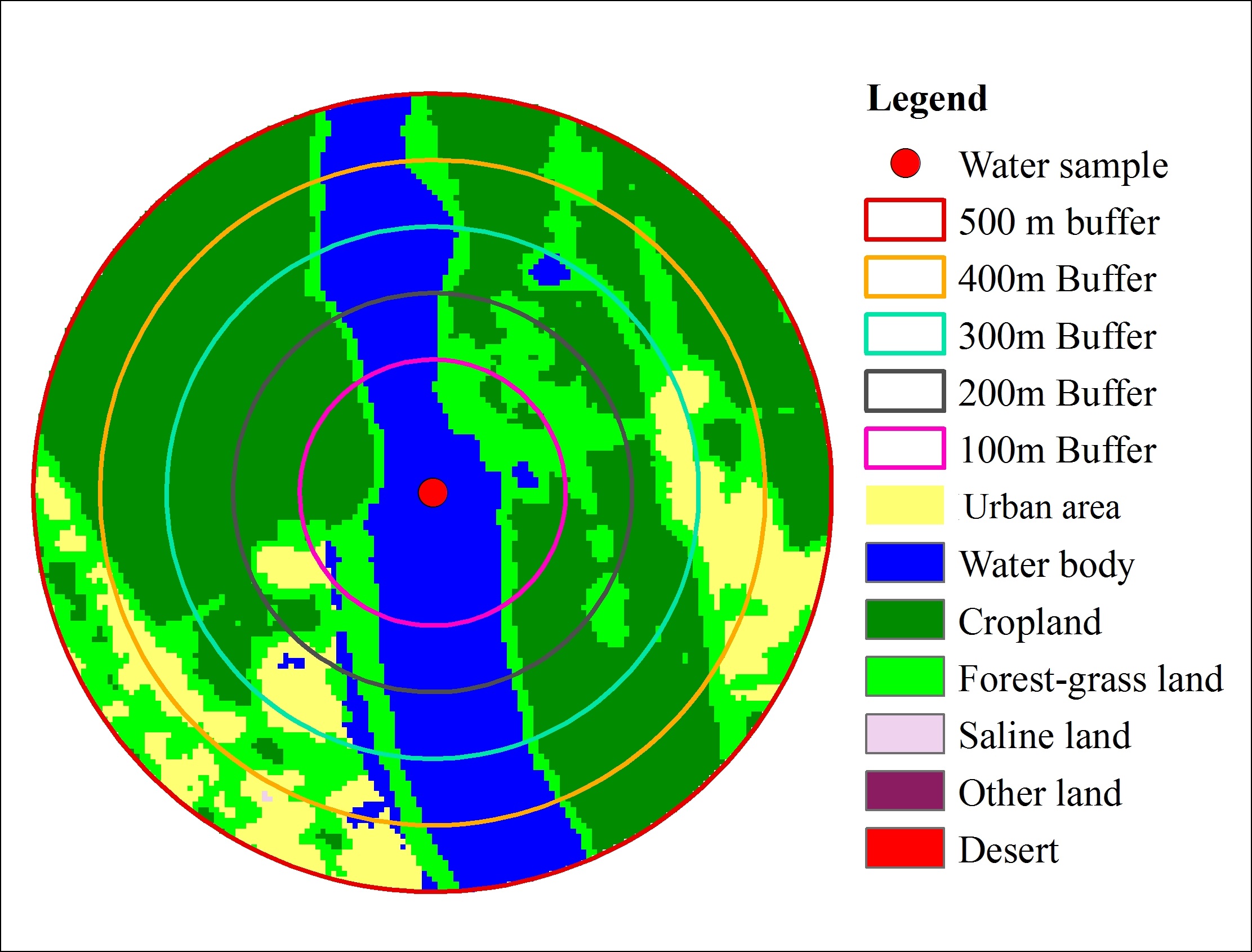


Fig. S2 Map of the buffer radius (Map by ArcGIS10.2.2 (http://www.esri.com/software/arcgis))
